# mTOR-Dependent Cell Proliferation in the Brain

**DOI:** 10.1155/2017/7082696

**Published:** 2017-11-13

**Authors:** Larisa Ryskalin, Gloria Lazzeri, Marina Flaibani, Francesca Biagioni, Stefano Gambardella, Alessandro Frati, Francesco Fornai

**Affiliations:** ^1^Department of Translational Research and New Technologies in Medicine and Surgery, University of Pisa, Via Roma 55, 56126 Pisa, Italy; ^2^I.R.C.C.S. Neuromed, Via Atinense 18, Pozzilli, 86077 Isernia, Italy

## Abstract

The mammalian Target of Rapamycin (mTOR) is a molecular complex equipped with kinase activity which controls cell viability being key in the PI3K/PTEN/Akt pathway. mTOR acts by integrating a number of environmental stimuli to regulate cell growth, proliferation, autophagy, and protein synthesis. These effects are based on the modulation of different metabolic pathways. Upregulation of mTOR associates with various pathological conditions, such as obesity, neurodegeneration, and brain tumors. This is the case of high-grade gliomas with a high propensity to proliferation and tissue invasion. Glioblastoma Multiforme (GBM) is a WHO grade IV malignant, aggressive, and lethal glioma. To date, a few treatments are available although the outcome of GBM patients remains poor. Experimental and pathological findings suggest that mTOR upregulation plays a major role in determining an aggressive phenotype, thus determining relapse and chemoresistance. Among several activities, mTOR-induced autophagy suppression is key in GBM malignancy. In this article, we discuss recent evidence about mTOR signaling and its role in normal brain development and pathological conditions, with a special emphasis on its role in GBM.

## 1. Introductory Statement: Molecular Structure and Functions of mTOR

The mammalian Target of Rapamycin (mTOR) is a 289-kDa serine/threonine kinase which belongs to the PI3K-related kinase (PIKK) family. It was originally discovered in yeast in the early 1990s. mTOR is a highly evolutionarily preserved kinase and it is ubiquitously expressed in all eukaryotic cell types including neural cells [1]. This protein is the target of a molecule named rapamycin, a lipophilic macrolide compound produced by the bacterium* Streptomyces hygroscopicus*, which was isolated for the first time in the 1970s in a soil sample from Easter Island (*Rapa Nui *in Polynesian) [2, 3].

This kinase plays a pivotal role in cell growth and metabolism acting as a key sensor and integrator of a variety of intra- and extracellular stimuli encompassing nutrients, growth factors, and energetic status and it represents a downstream substrate of PI3K/PTEN/Akt pathway, which controls protein synthesis and gene transcription, proliferation, and motility [4–6].

**Figure 1:**
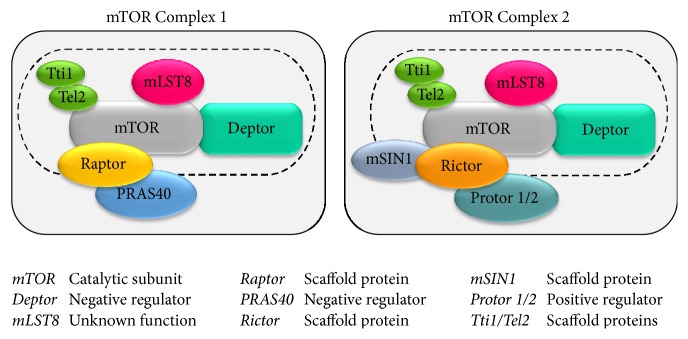
*mTOR structure and components*. In mammals, the mTOR kinase interacts with several proteins to form two functionally distinct multiprotein complexes, namely, mTOR Complex 1 (mTORC1) and mTOR Complex 2 (mTORC2). The dashed line indicates the components shared between mTORC1 and mTORC1.

**Figure 2:**
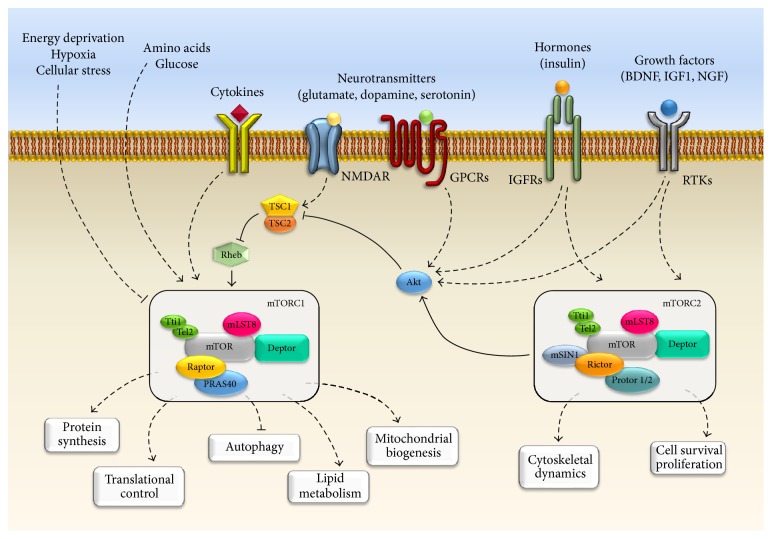
*mTOR signaling in neurons*. The cartoon summarizes the main pathways placed both upstream and downstream to mTOR. The activation of mTORC1 is elicited by a variety of upstream signaling molecules such as growth factors (e.g., BDNF, NGF, and IGF1), hormones (e.g., insulin), and amino acids and neurotransmitters (e.g., glutamate) via the stimulation of various receptors. These include RTKs (Receptor tyrosine kinases), GPCRs (G-protein coupled receptors), channel receptors, and cytokines receptors. Conversely, stimulation of NMDA receptor decreases mTOR activity. Even hypoxia and energy defect enhance TSC complex activity, which in turn leads to mTOR inhibition. The main downstream effects of mTORC1 are reported. Classically mTORC1 activates protein synthesis, translation, lipid biogenesis, and mitochondrial biogenesis, while autophagy is under the negative control of mTORC1. In contrast, mTORC2 is not sensitive to nutrients and it is mostly activated by growth factors and hormones to control cell survival and cytoskeletal organization.

In mammals, the mTOR catalytic subunit interacts with several proteins to form two big, functionally distinct, multiprotein complexes known as mTORC1 (mTOR Complex 1) and mTORC2 (mTOR Complex 2) [7–10] (Figure 1). Complex 1 (mTORC1) is composed of the catalytic subunit mTOR, the regulatory protein Raptor, and the proteins PRAS40, Deptor, and mLST8. In particular Raptor (regulatory associated protein of mTOR) acts as a scaffold protein for recruiting the mTOR substrates [11–13]. mTORC1 also associates with PRAS40 (proline-rich Akt substrate of 40 kDa) and Deptor (DEP-domain-containing mTOR-interacting protein), which act as negative regulators of the complex by inhibiting the binding of its substrates [14–16]. In addition, this complex contains the mLST8 protein (mammalian lethal with Sec13 protein 8; also known as G*β*L); however, the function of this last component is still unclear [15–17]. On the other hand, Complex 2 (mTORC2) encompasses six different components including mTOR, mLST8, Deptor, Rictor, mSIN1, and Protor 1/2 [16, 18–20]. In particular, the catalytic subunits mTOR and mLST8 and the negative regulator Deptor represent a common feature of both mTOR Complexes 1 and 2. Moreover, mTORC2 is equipped with the scaffold protein Rictor (rapamycin-insensitive companion of mTOR), the protein mSIN1 (mammalian stress-activated map kinase-interacting protein 1), which helps the complex assembly, and Protor 1/2 (so called protein observed with Rictor-1 and Rictor-2) [18–22]. In 2010 Kaizuka and colleagues [23] identified two other proteins (Tti1 and Tel2) which assemble with both mTORC1 and mTORC2 complex. These two mTOR-interacting proteins have been found to be essential for the stability and the assembly of this multiprotein complex. In fact, authors demonstrated that the knockdown of either Tti1 or Tel2 causes the disruption of mTOR functional activity [23]. The differences between mTORC1 and mTORC2 are not merely based on their protein components since these complexes are involved in the regulation of different major cellular processes (Figure 1). In particular, mTORC1 is mostly involved in cell growth and proliferation in response to energetic and nutritional conditions [24] (Figure 2). Nutrient availability (such as amino acids), cellular energy status (oxygen and AMP/ATP ratio), growth factors, and other extracellular and environmental stimuli may activate mTORC1, which in turn acts on protein synthesis through a wide number of downstream substrates and in particular on the molecules of the translational machinery responsible for the recruitment of mRNA (4E-BP1, p7026K, and S6 ribosomal protein) [13, 25, 26]. mTORC1 is also involved in regulating lipid biogenesis which is necessary for cell membrane generation and therefore cell growth and proliferation. In fact, it has been demonstrated that mTORC1 positively regulates the activity of two transcriptional factor, SREBP1 (sterol regulatory element binding protein 1), and PPAR*γ* (peroxisome proliferator-activated receptor *γ*), which are involved in controlling the expression of genes coding for proteins linked to lipid and cholesterol homeostasis [27, 28]. Moreover, mTORC1 regulates metabolism and mitochondrial biogenesis by modulating the transcriptional activity of the nuclear cofactor PGC1-*α* (PPRA*γ* coactivator 1) [29, 30]. Furthermore, mTORC1 is a negative modulator of autophagy, the main way of removing and recycling misfolded or long-lived macromolecules, and even entire damaged organelles (mitochondria, ribosomes, and endoplasmic reticulum) [31–35]. This latter process works in baseline conditions but can be either up- or downregulated depending upon specific needs. When a defect in the autophagy pathway occurs, a variety of cell mechanisms are altered and several consequences may be produced. In the last decade, the impairment of autophagy was related to a wide spectrum of human diseases including type II diabetes, neurodegenerative conditions and tumors as well [1, 36–38]. In contrast, mTORC2 is insensitive to nutrients and it responds mostly to growth factors and hormones to control actin cytoskeleton organization by phosphorylating several kinases such as Akt, SGK1, and PKC*α* [1, 10] (Figure 2). When compared to mTORC1, the function of mTORC2 is less explored. The dearth of knowledge about mTORC2 signaling pathways is mainly due to lethality caused by the deletion of mTORC2 components during embryonic development. We also lack specific mTORC2 inhibitors.

Only mTORC1 is sensitive to rapamycin [7]. This natural compound, produced by* Streptomyces hygroscopicus* bacteria, and its analogs (rapalogs) represent allosteric inhibitors which prevent mTORC1 recruitment of the mTOR catalytic subunit, leaving intact the mTORC2 activity [2, 3, 39–43]. Originally mTORC2 was thought to be a rapamycin-insensitive companion of mTORC1 [18, 21]. However, further studies demonstrated that, at least in some cell line, a prolonged rapamycin administration may inhibit mTORC2 function as well [44].

## 2. mTOR Signaling Pathway in Neurons

In cells, mTOR activation requires the integration of a variety of stimuli which in turn lead to several biochemical downstream reactions governing cell growth and metabolism. In neurons, major mTOR upstream inputs include amino acids (e.g., leucine and arginine) [45, 46], neurotrophic growth factors, and neurotransmitters [47]. In fact, mTOR is activated by a large number of growth factors encompassing BDNF (brain-derived neurotrophic factor), IGF1 (insulin-like growth factor 1), VEGF (vascular endothelial growth factor), CNTF (ciliary neurotrophic factor), and NRG-1 (neuregulin-1), all of them stimulating their specific tyrosine kinase (RTKs) receptor [47–50]. Most pathways which activate mTORC1 converge in inhibiting the TSC1-TSC2 (hamartin-tuberin) complex, a heterodimer which, in turn, is a strong endogenous mTOR inhibitor [51], while amino acids activate mTORC1 independently from TSC complex (Figure 2). In particular, mTORC1 activation is elicited by the inactivation TSC complex via its phosphorylation on specific sites through different kinases such as canonical Akt, RSK (ribosomal S6 kinases), or even IKKB (I*κ*B kinase *β*) [51].

Moreover, several molecules other than neurotrophic factors, such as guidance molecules, may either activate or inhibit mTOR activity. For instance, previous studies demonstrated that ephrin (Eph) is essential for axonal guidance by its inhibitory role on neuronal mTORC1 [52]. On the other hand, mTORC1 is activated by the reelin, an extracellular matrix protein, in order to regulate dendritic growth and branching [53, 54]. Again, many G-protein coupled receptors (GPCPs) have been reported to activate mTOR signaling in neuronal cells such as glutamate metabotropic mGlu1/5, AMPA, dopamine D_1_ and D_3_, GABA_B_, and serotonin 5-HT_6_ receptors [47, 55–59]. Conversely, the activation of the glutamate NMDA receptor reduces intracellular arginine, which decreases mTORC1 activity [60, 61]. Accordingly, the NMDA receptor antagonist ketamine was found to quickly activate the mTORC1 pathway [62, 63]. This latter effect leads to synaptogenesis in the prefrontal cortex of rat [62, 63]. In contrast only few data are available about upstream and downstream mechanisms bound to mTORC2 in neurons. For instance, mTORC2 activation may be induced by neurotrophic growth factors rather than nutrients, while the inhibition of its activity seems to be related to mTORC1 overactivation [64, 65] (Figure 2).

## 3. mTOR-Dependent Brain Development and Adult Neurogenesis

**Figure 3:**
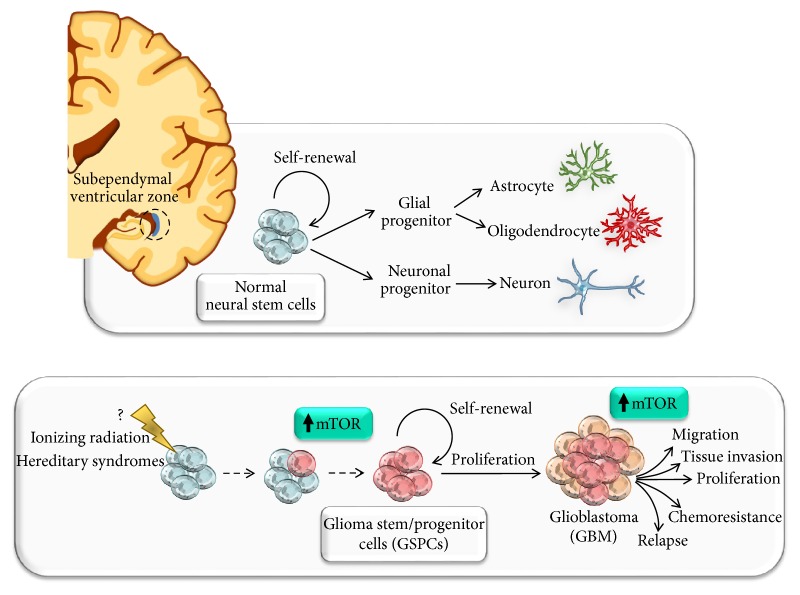
*The role of mTOR signaling in Glioma Stem/Progenitor Cells (GSPCs)*. mTOR is involved in neural stem cells (NSCs) proliferation, migration, differentiation, axonal and dendritic development, synaptic plasticity, learning, and memory storage. Aberrant mTOR signaling alters neural development and produces brain malformations in a wide spectrum of neurological disorders including neurodegeneration and brain tumors. In fact, mTOR governs the proliferation and maintenance of NSCs within normal CNS. These stem cell niches are placed within the subependymal ventricular zone of cornu temporalis nearby the cornu ammonis and dentatus gyrus of hippocampus. Several reports demonstrate that a fine spatiotemporal tuning of mTOR expression in the forebrain is essential for normal brain physiology and development. Thus, enhanced activation of mTOR may lead to brain malformation and neurodevelopmental disorders. In GBM, Glioma Stem/Progenitor Cells (GSPCs), which represent the amplification of normal stem cell niches, have been reported to support microvascular proliferation and to promote infiltration into the surrounding tissues. Moreover, these cells are key for GBM progression, radio- and chemoresistance and recurrence. Among several pathways which have been implicated in the maintenance and viability of GSPCs population, the marked upregulation of mTOR is key in fostering cancer stem cells self-renewal and malignant phenotype.

As occurring in all peripheral tissue, even in the central nervous system (CNS), the mTOR pathway is involved in many physiological functions such as cell growth, proliferation, migration, protein synthesis, and transcription [1, 4, 6, 13, 25, 66, 67]. Among these pathways, transcriptional control and protein synthesis are fundamental in the adult CNS due to their key role in synaptic plasticity. In this way, modification in those neuronal circuitries may alter learning and memory. This calls for an in-depth experimental analysis of the effects induced by long-term mTORC1 inhibition which may be induced therapeutically by rapamycin or rapalogs. In fact, apart from therapeutic effects, or systemic short-term side effects, there is no study which specifically addresses how the brain is modified by a long-term mTORC1 inhibition. This calls for ad hoc experiments designed to evaluate potential behavioural or movement alterations and their potential neurochemical and neuroanatomical basis. Remarkably, mTORC1-induced plastic events are dependent on novel protein synthesis. In fact, mTORC1 participates in early CNS development by regulating maintenance of neural stem cells, neuronal differentiation, migration, and axonal and dendritic development [68–84] (Figure 3). Even adult neurogenesis, which persists in adult mammals, has been shown to be an mTOR-dependent cellular process. This occurs in specific adult brain regions known as neurogenic niches which are the source of adult neuronal stem cells [77, 85–89]. Thus, it is not surprising that disruption or dysregulation of mTOR signaling pathway results in abnormalities of neuronal development and brain malformations causing a wide spectrum of brain disorders such as autism, seizures, and mental retardation syndromes [59, 78, 90–93]. The effects of mTORC1 disruption in the adult brain need consistent experimental efforts. Among all the components which constitute mTOR complexes, those which have been examined more in depth in the nervous system are the mTOR catalytic subunit, Raptor, Rictor, and mSIN1. Several reports have showed that mTOR is essential for normal brain physiology and development [17, 94–98]. In particular, the first evidence came from a genetic screening which led to the isolation of selective mutations affecting the early telencephalon patterning [99]. One of the four isolated mutants, named* flat-top*, revealed a specific lack of the telencephalon due to a disrupted signaling pathway which regulates telencephalic primordia expansion and regionalization. Noteworthy these* flat-top* mutant mice, owning a single nucleotide intronic mutation which resulted in aberrant splicing and decreased mTOR activity, showed a failure of telencephalic vesicles progression [94]. Moreover, it has been demonstrated that mTOR null mice die shortly after implantation at early embryonic stages (E6.5–7.5), even before the active proliferation of neural progenitors, which start generating cortical neurons from embryonic day 10 to day 17 [17, 95, 96]. Whereas the complete deletion of mTOR results in the lack of telencephalon and early death of mice embryos, it has been recently demonstrated that even overactivation of mTOR leads to pathological alterations in brain development. For instance, mutant mice carrying mTOR gain-of-function mutations (CAG-mTOR^SL1+IT^/+; Emx1^cre/+^) at early embryonic stages showed an atrophic cerebral cortex, while the mTOR overactivation in postmitotic neurons from late embryonic stages or postnatal period leads to cortical hypertrophy and severe epileptic seizures [78, 100]. Thus, a fine spatiotemporal tuning of mTOR expression in the forebrain is likely to be key for preserving CNS development.

Again, the complete ablation of mTOR components other than the catalytic subunit results in embryonic lethality in mice [17, 101]. For instance, mice embryos deficient for Raptor or mLST8 die at early developmental stages, around E6.5 and E10.5, respectively, suggesting that these components are essential for mTOR proper function [17]. Consistent with previous reports [17, 94–96], it has been demonstrated that the disruption of the mTORC2 specific component Rictor is lethal at early embryonic stages in mice. When compared with their wild-type littermates, Rictor null embryos showed growth arrest between E9.5 and E10.5 and then die by E11.5 [101]. Remarkably, a very recent study showed that silencing Rictor gene expression by RNAi (RNA interference) does not enable mouse one-cell stage embryo to enter into the two-cell stage normally [102]. In particular, the lack of Rictor expression dramatically decreased the egg cleavage of mouse one-cell stage embryo, which was blocked at G2 phase. Therefore, these data suggest that Rictor-mTORC2/Akt1 pathway is essential for early mitotic division in early embryos [102].

Since knockout (KO) mice with a complete loss of mTOR showed a severe phenotype with specific defect in telencephalon formation and death during early/mid gestation [17, 78, 95, 96, 99, 101, 102], conditional knockout (CKO) mice were fundamental to elucidate the functional role of mTOR in the brain [77, 78, 87, 88]. Recent CKO mouse studies have demonstrated that the disruption of mTOR signaling in the brain causes alteration in the homeostasis of neural progenitors due to unbalanced self-renewal and differentiation processes [76–78, 94, 96]. Remarkably, Ka et al. [77] showed that in conditional mTOR knockout mice (mTOR^loxP/loxP^; Nestin-cre) the deletion of mTOR in neural progenitors reduces neuronal layers within the developing cerebral cortex. Thus the cerebral cortex appeared to be markedly reduced in both weight and thickness at pathological examination. Moreover, recent studies found that mTOR activity was impaired in the NSCs of aged brain. In particular, mTOR activity was decreased within NSC niches in adult and aging forebrain [86, 103]. Accordingly, ketamine-induced mTOR signaling activation increases adult NSCs proliferation in aged mice thus reducing age-associated decline in neurogenesis [103]. This evidence strongly suggests that mTOR-mediated signaling is key in the maintenance of NSCs and affects neuronal differentiation. Again, Zhang et al. [81] showed that mTOR signaling amplifies adult NSCs and progenitor cells which are crucial during hippocampal neurogenesis, which is associated with spatial learning and memory. In fact, within adult subgranular zone (SGZ) the rate of neurogenesis is regulated by a gene silencer (enhancer of zeste homolog 2, Ezh2), which promotes the amplification of active NSCs and progenitor cells acting through the Pten-Akt-mTOR pathway. In particular, Ezh2 suppresses Pten expression and promotes the activation of Akt-mTOR [81].* In vivo* Exh2 deletion decreases mTOR activity and reduces proliferation of progenitors cells, which leads to impaired learning and memory in Ezh2-null mice [81].

In keeping with the role of mTOR in proliferation and migration of neural precursors, Lafourcade et al. [104] showed that mTOR hyperactivation in neural progenitor cells (NPCs) leads to migratory heterotopia, ectopic neuron placement, and abnormal neuronal morphogenesis. Increases in mTOR activity in neonatal NPCs of the subventricular zone (SVZ) of wild-type mice by electroporating a constitutively active Rheb-encoding vector (Rheb^CA^), an mTOR positive upstream regulator, cause migratory heterotopia in the rostral migratory stream (RMS) and olfactory bulb (OB). Notably, these effects were prevented by rapamycin administration, thus validating mTOR involvement in these neuronal morphogenesis defects [104]. Migratory heterotopia at cortical level represents a major mechanism of epileptogenesis. Remarkably, very recent manuscripts emphasized the role of mTOR upregulation and reduced autophagy as causal mechanisms in epileptogenesis and epilepsy-induce neuronal damage [105].

Thus, mTOR plays a specific role within CNS for normal development which encompasses neurite elongation and branching, dendritic spine formation, synaptic consolidation and plasticity, memory storage, and cognition.

Some findings regarding dendritic and axonal growth are sometimes contradictory. This may be due to variability of* in vitro* models with minor differences in culture conditions [73, 74, 83, 106, 107]. Although further studies are needed* in vivo* in order to confirm the role of mTOR in development and neurogenesis the prevalent evidence indicates that mTOR is crucial for synaptic plasticity [70–72, 75, 108–110]. For instance, recent studies demonstrate than mTOR contributes to hippocampal synaptic plasticity strengthening long-term potentiation (L-LTP) [68, 70, 71, 75]. In Tsc2+/− mice the overactivation of hippocampal mTOR leads to abnormal hippocampal LTP in the CA1 region [72]. In particular, the reduced threshold for hippocampal LTP produces abnormal learning. These effects were reversed by administering the mTOR inhibitor rapamycin [72].

## 4. mTOR Related Neurological Disorders and Brain Tumors

Over the past decades, Akt-mTOR signaling has increasingly garnered a central role in regulating several molecular and biochemical pathways which are known to be altered in a variety of pathological conditions encompassing obesity, cardiovascular diseases, hypertension, type II diabetes, neurodegeneration, and brain tumors [37, 38, 66, 67, 111]. In the CNS a wide spectrum of psychiatric, neurological, and neurodegenerative disorders has been related to deregulated mTOR signaling pathway. Several mutations in mTOR regulatory genes (e.g., TSC1, TSC2, LYK5/STRADA, AKT3, and DEPDC5) enhance activation of mTOR and lead to brain malformation and neurodevelopmental disorders. This is the case of Tuberous Sclerosis Complex (TSC), hemimegalencephaly (HME), focal cortical dysplasia (FCD), Pretzel syndrome (Ps), and familial focal epilepsy with variable foci (FFEVF) [112–117]. Noteworthy, administration of mTOR inhibitors is frequently beneficial for treating some neurological alterations such as epilepsy, autism, and learning disabilities [118–123].

Among primary brain tumors the most common are gliomas which account for 80% of malignant CNS tumors. The majority of gliomas (about 76%) are astrocytoma [124, 125]. Several human studies provided strong evidence on the key role of an altered mTOR signaling pathway in low-grade astrocytoma, such as subependymal giant cell astrocytoma (SEGA), a rare, slow-growing benign tumor (WHO grade I) which mainly occurs in young patients [126, 127]. Based on this evidence, pharmacological inhibition of mTOR with sirolimus and everolimus was provided [128, 129] and, in 2010, everolimus was approved by FDA as an alternative to surgical resection for TSC-associated SEGA patients [130–133]. Again, several reports have found that mTOR pathway is frequently activated in other low-grade astrocytomas such as pediatric low-grade glioma (PLGG) and pilocytic astrocytoma (PA) [134, 135], while to date there are only few morphological studies regarding the role of mTOR in anaplastic astrocytoma (WHO grade III glioma) [136–138]. In contrast, there is increasing evidence of mTOR upregulation in both experimental and human Glioblastoma Multiforme (GBM). As witnessed by multiple experimental and neuropathological findings [139–141], mTOR upregulation is key in developing GBM aggressive phenotype [142–145].

## 5. mTOR Upregulation as a Key to Understand the Neurobiology of GBM

The biology of glioblastoma is characterized by prominent proliferation, active invasiveness, and rich angiogenesis, mainly due to highly mutated and/or deregulated signaling pathways within the tumor [146, 147]. Growing knowledge of these signaling pathways improved our understanding of the biology and clinical behaviour of GBM. Remarkably, several reports show that glioblastoma is more resistant to apoptosis-inducing therapies, rather than autophagy-inducing therapies [148, 149]. GBM resistance to conventional proapoptotic chemotherapy and radiotherapy results from changes at the genomic, transcriptional, and posttranscriptional level in key molecules involved in mitogenic signaling, mostly in the PI3K/PTEN/Akt axis, in tyrosine-kinases receptors (RTKs) and their ligands, and in regulatory molecules and effectors of the apoptotic cell death pathway [148, 150].

Several aberrant antiapoptotic signals conferring intrinsic resistance to conventional and targeted anticancer therapies have been described in GBM [149]. For instance, up to 70% of GBM have an alteration of tumor suppressor gene PTEN [124, 148, 151]. This results in constitutive and increased activation of several downstream effectors of the signaling pathways controlled by PI3K, of which the most important identified to date is PTEN/PI3K/Akt/mTOR, which plays a critical role in the regulation of gene expression and prevention of apoptosis [139, 150].

GBM consists of heterogeneous populations of poorly differentiated tumor cells and it is characterized by a necrotic area that can present foci of micronecrosis surrounded by hypercellular zones, near to the normal tissue, of infiltrates derived from parenchymal tissue. These cells surrounding the necrotic foci are organized to form pseudopalisades with a configuration that is typically exclusive of glial tumors [152]. Intense microvascular proliferation and infiltration into the surrounding tissues occur [153]. This is supported by the presence of niches containing stem/progenitor cells of GBM.

In the last decade, consistent evidence indicates that within the tumors (mostly hematopoietic and solid tumors) there is a fraction of cells, the cancer stem cells (CSCs), which share characteristics with normal stem cells, such as self-renewal potential and maintained proliferation, and thus can initiate and sustain the tumor [154–156]. These cells also have the ability to propagate the tumor in an orthotopic xenograft transplantation model, when compared with the nontumorigenic cells within the tumor bulk [157]. This theory, known as “The Cancer Stem Cell Hypothesis,” has been proposed for the first time by Reya and colleagues in 2001. Nowadays, such fraction of cells has also been identified in GBM, known as Glioma Stem/Progenitor Cells (GSPCs) [158, 159]. In particular, these cells, which represent the amplification of normal stem cell niches placed within the subependymal ventricular zone of cornu temporalis nearby the cornu ammonis and dentatus gyrus of hippocampus, have been reported to support microvascular proliferation and to promote infiltration into the surrounding tissues [160–162]. It is also very likely that GSPCs are responsible for GBM progression, radio- and chemoresistance, and recurrence [159, 163, 164]. Several pathways have been implicated in the regulation and maintenance of GBM cancer stem cells pool. Among them, the most important is the PI3K/Akt/mTOR which, as aforementioned, needs to be properly and tightly regulated in order to ensure a wide number of physiological processes (i.e., proliferation, metabolism, survival, differentiation, and autophagy) [38]. In GSPCs the marked upregulation of mTOR has been related to increased proliferation, invasiveness, and resistance to standard treatments (Figure 3). Thus, aberrant Akt/mTOR signaling pathway strongly correlates with GBM malignancy and poor prognosis [142–145]. Growing evidence shed the light on the involvement of PTEN/Akt/PI3K/mTOR signaling in maintenance and viability of GSPCs population [141–145, 165, 166]. For instance, Garros-Regulez et al. [145] have demonstrated that pharmacological inhibition of mTOR in glioma stem cells (GSCs) causes a reduction in the expression of SOX2 and SOX9, which are required for neural stem cell maintenance, thus decreasing GSCs self-renewal potential and proliferation. Moreover, it has been demonstrated that the lack of differentiation of GSPCs is related to mTOR-dependent autophagy inhibition [142, 158, 167–171]. Among several pathways which are modulated through mTOR signaling, this molecular complex plays a major role as an endogenous autophagy inhibitor [172]. Notably, autophagy depression represents a hallmark of GBM, as confirmed by several pathological studies carried out on human high-grade astrocytoma samples [140, 168, 173, 174]. Several biochemical findings demonstrate the remarkable effects of autophagy activators as powerful inducers of cell differentiation, with a strong prevalence towards neuronal phenotypes [142, 171, 175–180]. Remarkably, autophagy activation has demonstrated a beneficial effect in a variety of gliomas [172–182]. Again, GSPCs cell migration and infiltration, which represent key mechanism for GBM progression and infiltration within the surrounding normal brain tissue, rely on mTOR upregulation and autophagy suppression [165, 171]. Experimental evidence was recently provided by our group showing that the mTOR inhibitor rapamycin produces a remarkable suppression of GBM cell proliferation both* in vitro* and* in vivo* in xenografts. In detail, rapamycin produces a time- and dose-dependent suppression of GBM cell proliferation when tested both in GBM cell lines and primary cell cultures from GBM patients [168]. This effect is remarkably similar to U87MG cell lines and patient-derived primary cell cultures and is not accompanied with a cytotoxic effect. It is rather a suppression of cell growth, as shown by cytofluorometry and Ki-67 immunostaining, which is responsible for antiproliferative activity induced by mTOR inhibition. The rapamycin-administered GBM cells possess an increase in autophagy (ATG) vacuoles and develop a progressive differentiation towards a neuron-like phenotype [168]. Remarkably, the mTOR suppression induced by rapamycin is not accompanied by increased in pAkt/Akt ratio ruling out the risk of a rebound Akt overactivation. It is worth mentioning that the volume suppression induced by rapamycin* in vivo* in xenografted GBM appears to surpass the magnitude of antiproliferative activity. In fact, the tumor volume which is measured following rapamycin administration is way reduced compared with tumor volume which was already present before rapamycin was administered [183]. Since such an effect occurs in the absence of any apoptotic, necrotic, or gliotic area, what is the fate of the previous volume filled with GBM since following rapamycin this appears to be regularly shaped as specific CNS tissue remains to be established [183]. Specific experiments aimed at deciphering a potential phenotypic rescue of malignant GBM cells back to differentiated neural tissue are currently in progress in our lab. In fact, a few months ago we found that rapamycin while inhibiting cell proliferation produced a massive stemness regression in vitro as measured by classic antigens. This occurs in association with the expression of early and late neural differentiation markers, such as NeuroD, beta-III tubulin, and NeuN, respectively. Remarkably, this phenotypic shift is concomitant with suppression of GBM cell migration [171]. All these events are backed up by specific epigenetic effects of rapamycin on stemness and differentiation genes. Despite the innumerous cascades which reside under the modulation of mTOR, the ATG machinery appears to be the most likely to counteract GBM progression. In fact, the very same doses of rapamycin produce a remarkable increase in ATG vacuoles and promote the merging of the proteasome pathway within ATG vacuoles to produce a fusion organelle named autophagoproteasome [184]. The shuttling of proteasome within ATG vacuoles was recently demonstrated to depend on the recognition of the ATG protein p62 which translocate proteasome components within LC3-positive ATG vacuoles [185]. While low doses of mTOR inhibition increase the ATG compared with proteasome activity, high doses of rapamycin, way in excess to those required to inhibit GBM proliferation, produce a prevalence of proteasome over ATG activity. The ATG-inducing doses of rapamycin range between 5 and 10 nM which correspond to the amount of rapamycin considered to be in the therapeutic range in humans as an immunosuppressant [3, 186]. The very same range of doses fills the dose response curve for the therapeutic effects of rapamycin we measured in experimental GBM [168, 171]. It is noteworthy that, as it was demonstrated* in vivo* [183], these doses of rapamycin are not effective in producing apoptosis [168] and do not exert any cytotoxic effects in GBM cells [171]. Altogether these data pose the intriguing hypothesis that mTOR inhibition may remodel the anaplastic GBM cell population stimulating “*a rebours,*” a phenotypic differentiation shift. In other words, inhibiting mTOR in glioblastoma may suppress an overactive neural stem cell proliferation readdressing this exuberant cell population towards the original phenotypes. Dedicated experiments are needed to provide definite validation of these early data and to establish the concomitant alterations which may occur in the healthy areas of the CNS under the effects of prolonged mTOR inhibition.

Nowadays a number of clinical trials are ongoing to evaluate the effects of mTOR inhibition with rapamycin (or rapalogs) based on the exciting results obtained both* in vitro* in glioblastoma cell lines and in preclinical* in vivo* models of GBM. Nonetheless, conflicting data still exist on rapamycin and rapalogs treatments for high-grade astrocytomas [187, 188]. For instance, it has been reported that rapamycin inhibition of mTORC1-mediated S6K phosphorylation causes the block of a negative feedback loop which in turn promotes the activation of mitogenic pathways such as PI3K/Akt and Ras/MEK/ERK [189]. The activation of these latter pathways causes an mTORC2 and Akt hyperactivation through other feedback loops, which may contribute to GBM metabolic reprogramming and drug resistance [190–192]. Since mTOR inhibition promotes hyperactivation of the PI3K/Akt pathway, which could limit the drug treatment efficacy, most of current trials take advantage of combined therapies by administering dual PI3K-mTOR inhibitor or new generation compounds such as ATP-competitive mTOR inhibitors, which inhibit both mTORC1 and mTORC2 [166, 191–198]. However, the extent of the crosstalk between these molecular complexes is not fully understood, since to date only few upstream effectors and downstream substrates of mTORC2 have been identified. A careful analysis of these report demonstrate that such a feedback activation of Akt under the effects of mTOR inhibition is indeed very rare in human patients and it is more frequent to observe the opposite effect [168].

## 6. Summary

The mammalian Target of Rapamycin (mTOR) is a multiprotein complex equipped with kinase activity which belongs to the serine/threonine protein kinase (PI3K-related kinase, PIKK) family. The mTOR catalytic subunit, which nucleates two functionally distinct complexes (mTORC1 and mTORC2), is a key mediator of the PI3K/Akt/mTOR signaling which controls cell growth, proliferation, and metabolism. mTOR signaling is essential for normal development and physiology of nervous system. In fact, several physiological brain functions depend on mTOR activation. Increasing evidence demonstrates that mTOR is critical for maintaining the stemness of neural stem cells, neuronal differentiation and migration, and axonal and dendritic development. In addition, in adult brain synaptic plasticity, learning and memory storage require a fine modulation of mTOR activity. Therefore, dysregulation of this pathway has been implicated in numerous pathological conditions encompassing cancer, obesity, type II diabetes, neurological and psychiatric disorders, neurodegeneration and brain tumors, and mostly astrocytomas.

As witnessed by multiple experimental and neuropathological findings, mTOR upregulation plays a major role in the development of the aggressive phenotype of glioblastoma (GBM, WHO grade IV astrocytoma), thus influencing prognosis and determining response to therapies. Although standard treatment options for GBM patients provide maximal surgical resection, in combination with chemotherapy (with temozolomide, TMZ) and radiotherapy, it becomes increasingly clear that modulation of mTOR activity represents an important molecular target. It should be mentioned that mTOR inhibitors have not demonstrated therapeutic potential in clinical trials against glioblastoma so far. An intense clinical investigation is going on with approximately twenty current clinical studies using mTOR inhibitors for the treatment of gliomas. However, phase II studies reported no efficacy of temsirolimus in combination with temozolomide, sorafenib, bevacizumab, or erlotinib in recurrent glioblastoma [199]. In any case, understanding the role of mTOR in brain development and neurogenesis will contribute to elucidate the pathological mechanisms of several neurological, neurodevelopmental, and neurodegenerative disorders.
